# Detection of Epstein‒Barr virus DNA methylation as tumor markers of nasopharyngeal carcinoma patients in saliva, oropharyngeal swab, oral swab, and mouthwash

**DOI:** 10.1002/mco2.673

**Published:** 2024-08-19

**Authors:** Xiao‐Hui Zheng, Xi‐Zhao Li, Cao‐Li Tang, Yu‐Meng Zhang, Ting Zhou, Xiao‐Jing Yang, Ying Liao, Yong‐Qiao He, Tong‐Min Wang, Wen‐Qiong Xue, Wei‐Hua Jia

**Affiliations:** ^1^ State Key Laboratory of Oncology in South China Guangdong Key Laboratory of Nasopharyngeal Carcinoma Diagnosis and Therapy Guangdong Provincial Clinical Research Center for Cancer Sun Yat‐Sen University Cancer Center Guangzhou China; ^2^ School of Public Health Sun Yat‐Sen University Guangzhou China

**Keywords:** Epstein‒Barr virus DNA methylation, nasopharyngeal carcinoma, oral swab, oropharyngeal swab, saliva

## Abstract

Saliva biopsy of nasopharyngeal carcinoma (NPC) has been developed in our latest study, indicating the application of oral sampling in NPC detection. Further exploration of the potential for self‐sampling from the oral cavity is necessary. A total of 907 various samples from oral cavity, including saliva (*n* = 262), oropharyngeal swabs (*n* = 250), oral swabs (*n* = 210), and mouthwash (*n* = 185), were collected. Epstein‒Barr virus (EBV) DNA methylation at the 12,420 bp CpG site in EBV genome from the repeat‐copy W promoter (Wp) region and at the 11,029 bp CpG site in the single‐copy C promoter (Cp) region were simultaneously detected in these samples. A significant increase in EBV methylation, no matter at Wp or Cp region, was found in all types of samples from NPC patients. However, EBV DNA methylation in saliva and oropharyngeal swab showed a better diagnostic performance in detecting NPC. The combination of these two sample types and two markers could help to improve the detection of NPC. Our study further explored the optimal self‐sampling methods and detection target in the detection of NPC and may facilitate the application of EBV DNA methylation detection in a home‐based large‐scale screening of NPC.

## Graphical abstract

In oral cavity, self‐sampling samples, including saliva, oropharyngeal swab, oral swab, and mouthwash, can all be done by donors themselves. In this study, EBV DNA methylation was detection and compared in these samples. Our study explored the optimal self‐sampling types and detection targets for the detection of NPC and may facilitate the application of EBV DNA methylation detection in a home‐based large‐scale screening of NPC.

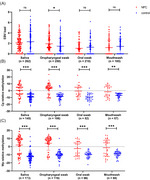

## 1 INTRODUCTION

Nasopharyngeal carcinoma (NPC) is tumor originating from the mucosal epithelium of the nasopharynx that is highly invasive and metastatic and is prevalent mainly in south China.^1^ Unfortunately, due to the insidious nature of the early symptoms, the majority of NPC patients are diagnosed at an advanced stage when they are first diagnosed.^2^ Therefore, early detection of NPC is of great importance.

Epstein‒Barr virus (EBV) is a herpesvirus that infects more than 90% of the world's population and is known to be associated with several cancers, including NPC, lymphoma, gastric cancer, and so on. EBV plays an important role in NPC tumorigenesis, and there are many studies on the oncogenic mechanism of EBV. The EBV‐encoded proteins LMP1 and LMP2A have the capability to induce abnormal modifications in host DNA methylation by influencing the activities of methylation transferases in host cells. This process leads to extensive methylation modifications in several tumor suppressor genes such as RASSF1A, P16, and DAPK1, resulting in decreased expression and silencing of critical signaling pathways, ultimately promoting tumorigenesis. Besides, the methylation modifications of EBV DNA also have a significant change with tumor occurrence. W promoter (Wp), C promoter (Cp), and Q promoter (Qp) mediate the transcriptional expression of the EBV latency protein epstein‐barr virus nuclear antigens (EBNAs). Wp is the first EBV latency promoter, and after a few days, the transcriptional activity of Wp is switched off, and Cp is activated and referred to as the major latency promoter. EBV in NPC is in latent phase II infection, when the transcriptional activity of both Cp and Wp is turned off and transcriptional expression of EBNA1 is mediated through Qp. The development of EBV latent state in NPC is closely related to the functional transitions of the three promoters. Moreover, changes in methylation modifications play an important role in the functional switch of promoters.^3^, ^4^

Various EBV‐related biomarkers, including serum antibodies and plasma nucleic acid indicators, have been used in NPC screening.^5^, ^6^, ^7^, ^8^, ^9^, ^10^, ^11^, ^12^, ^13^, ^14^, ^15^, ^16^, ^17^, ^18^ We previously focused on a brush sampling method directly from nasopharynx and measured several different types of biomarkers, including DNA load, miRNA, and DNA methylation from EBV, in nasopharyngeal brushing samples. These studies showed that they could serve as a promising method for the detection of NPC.^16^, ^19^, ^20^, ^21^ Furthermore, brush sampling without the guidance of endoscope, named blind brushing, has been explored.^22^, ^23^ Besides, to further promote the application of EBV‐based nucleic acid detection, an ultra‐sensitive amplification‐free EBV DNA quantification technology based on CRISPR/Cas12a system and droplet microfluidic technology was also designed in our recent study.^24^

It is widely acknowledged that self‐sampling will greatly promote cancer's early detection. There are two successful examples, including the stool‐based detection of colorectal cancer and the urine‐based detection of cervical cancer. As a non‐invasive method, saliva sampling could be performed by donors themselves. In our latest study, we first brought the potential of saliva biopsy of NPC. EBV DNA methylation in saliva has been found to have a significant increase in NPC patients. The sensitivity and specificity of detecting a single CpG site (11,029 bp) located in Cp region could reach more than 70% sensitivity and nearly 100% specificity. Besides, the methylated density of the CpG site was found to decrease below the cut off value (COV) in NPC patients after therapy, and increase above the COV after recurrence,^25^ indicating the potential application of EBV DNA methylation detection in samples from oral cavity.

In oral cavity, self‐sampling samples including saliva, oropharyngeal swab, oral swab, and mouthwash can all be done by donors themselves. Compared to saliva sampling, oropharyngeal swab, oral swab, and mouthwash methods are easier and more convenient. However, the effectiveness of these oral cavity sampling methods for NPC detection is still under investigation. Therefore, detection of EBV DNA methylation was further conducted, and in these samples the results were compared. The region containing the Wp included sequences that were repeated multiple times and excluded from the EBV methylation capture sequencing. According to our previous study, the entire EBV genome was hypomethylated in control samples.^25^ It is inferred that there are also methylation modification differences in the Wp region. In theory, targeting the detection of intrinsic repetitive regions may enhance the detection rate. Therefore, this study simultaneously assessed the methylation levels 12,420 bp CpG site at the repeat‐copy Wp region and 11,029 bp CpG site in the single‐copy Cp region. Our results showed that EBV DNA methylation, no matter at Wp region or Cp region, all had a significant increase in all these sample types from NPC patients, and could be used as potential biomarkers of NPC. This research offers an attractive option for the non‐invasive identification of NPC, potentially contributing to home‐based screening methods in the future.

## 2 RESULTS

### 2.1 EBV DNA load and EBV DNA methylation were detected in saliva, oropharyngeal swab, oral swab, and mouthwash

**FIGURE 1:**
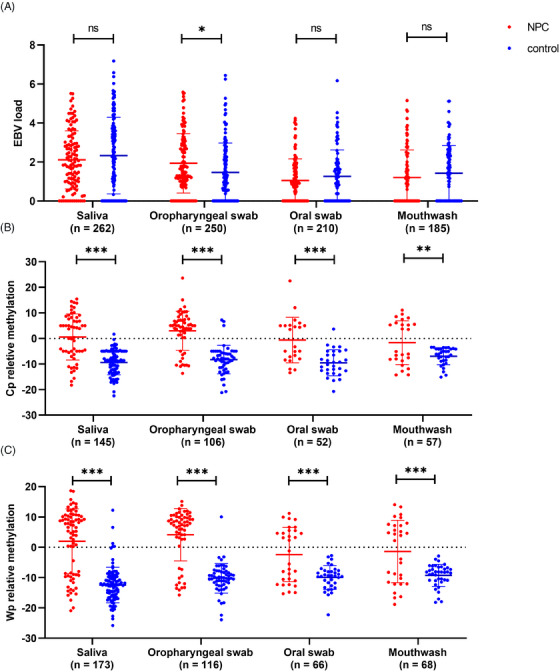
Epstein‒Barr virus (EBV) load and C promoter (Cp) and W promoter (Wp) methylation levels in saliva, oropharyngeal swab, oral swab, and mouthwash samples. The methylation levels of Cp (B) and Wp (C) all had a significant increase in nasopharyngeal carcinoma (NPC) group compared to controls in all these samples.

A total of 262 saliva, 210 oral swab, 250 oropharyngeal swab, and 185 mouthwash samples were collected and detected. The EBV DNA load and EBV DNA methylation were detected in these four types of samples. As shown in Table 1, the highest detection rate of EBV DNA load (79.39%) was observed in saliva, while the lowest detection (56.76%) was observed in mouthwash. The detection rate in oropharyngeal swab samples (74.40%) was similar to that in saliva samples. Quantitatively, the EBV load was highest in saliva samples. In oropharyngeal swab samples, the EBV load in the NPC group was slightly higher than that in the control group (1.50 vs. 1.16, *p* = 0.0154), while there were no statistically significant differences between groups in other types of samples (Table 1 and Figure 1A).

**TABLE 1:** Epstein‒Barr virus (EBV) DNA load in saliva, oropharyngeal swab, oral swab, and mouthwash samples.

	Saliva	Oropharyngeal swab	Oral swab	Mouthwash	*p*‐Value
EBV detection rate^a^ (%)					
NPC	104/118 (88.14)	97/119 (81.51)	77/119 (64.71)	52/97 (53.61)	<0.001
Control	104/144 (72.22)	89/131 (67.94)	58/91 (63.74)	53/88 (60.23)	0.2519
All	208/262 (79.39)	186/250 (74.40)	135/210 (64.29)	105/185 (56.76)	<0.001
EBV load [median (*P*_25_, *P*_75_)]					
NPC	1.94 (0.95, 3.00)	1.50 (0.91, 2.94)	0.90 (0.00, 1.68)	0.89 (0.00, 2.29)	<0.001
Control	2.27 (0.00, 3.78)	1.16 (0.00, 2.35)	1.00 (0.00, 1.89)	1.41 (0.00, 2.45)	<0.001
*p*‐Value	0.3058	0.0154	0.2256	0.2868	

Abbreviation: NPC, nasopharyngeal carcinoma.

^a^ Samples with EBV load >0 were considered positive.

Samples with positive EBV DNA load were further tested for EBV DNA methylation. Two CpG sites from two typical locations were simultaneously detected, including 12,420 CpG site in repeat‐copy Wp region and 11,029 CpG site in single‐copy Cp region. Among the 11,029 CpG site from Cp region, the highest and lowest detection were found in saliva (69.71%) and oral swab (38.52%), respectively. For the 12,420 CpG sites from Wp region, the highest detection rate was observed in saliva (83.17%), followed by mouthwash (64.76%) and oropharyngeal swab (62.37%). The lowest detection rate was observed in oral swab (48.89%). Overall, detection of CpG sites from Wp region had a higher detection rate (Table 2).

**TABLE 2:** Epstein‒Barr virus (EBV) DNA methylation in saliva, oropharyngeal swab, oral swab, and mouthwash samples.

	Saliva	Oropharyngeal swab	Oral swab	Mouthwash	*p*‐Value
Cp					
Detection rate (%)					
NPC	56/104 (53.85)	55/97 (56.70)	22/77 (28.57)	25/52 (48.08)	<0.001
Control	89/104 (85.58)	51/89 (57.30)	30/58 (51.72)	32/53 (60.38)	<0.001
All	145/208 (69.71)	106/186 (56.99)	52/135 (38.52)	57/105 (54.29)	<0.001
Relative methylation level [median(*P*_25_, *P*_75_)]					
NPC	3.78 (−5.13, 7.76)	5.00 (0.83, 7.10)	−4.12 (−7.79, 5.00)	−5.15 (−8.18, 5.55)	0.0482
Control	−8.90 (−13.14, −5.00)	−8.01 (−11.39, −5.29)	−9.76 (−12.63, −5.38)	−6.15 (−8.72, −4.16)	0.0669
*p*‐Value	<0.001	<0.001	<0.001	0.0064	
Wp					
Detection rate (%)					
NPC	75/104 (72.12)	59/97 (60.82)	31/77 (40.26)	30/52 (57.69)	<0.001
Control	98/104 (94.23)	57/89 (64.04)	35/58 (60.34)	38/53 (71.70)	<0.001
All	173/208 (83.17)	116/186 (62.37)	66/135 (48.89)	68/105 (64.76)	<0.001
Relative methylation level [median(*P*_25_, *P*_75_)]					
NPC	6.50 (−8.99, 10.14)	7.50 (2.91, 9.84)	‒1.20 (‒10.82, 5.06)	3.04 (−11.20, 7.15)	0.0080
Control	−12.48 (−16.06, −9.81)	‒10.16 (−12.22, ‒8.09)	‒9.47 (−12.25, −7.85)	‒8.23 (−10.88, −6.88)	0.0018
*p*‐Value	<0.001	<0.001	<0.001	<0.001	

Abbreviations: Cp, C promoter; NPC, nasopharyngeal carcinoma; Wp, W promoter.

The methylation level of Wp and Cp all had a significant increase in NPC group compared to controls in all these samples, including saliva, oropharyngeal swab, oral swab, and mouthwash (Table 2 and Figure 1B,C). However, in the NPC group, a higher methylation levels of Cp and Wp were observed in oropharyngeal swab (median = 5.00 and 7.50) and saliva (median = 3.78 and 6.50). There was no significant difference of EBV DNA methylation in these four samples types among different age and gender subgroup (Tables S1 and S2).

A qualitative analysis of the methylated type was further conducted. The quantitative methylation‐specific PCR (q‐MSP) detection results could be categorized into three types. Type M: only obtaining the CT value of the methylated product (CTm). Type U: only obtaining the CT value of the unmethylated product (CTu). Type MU: CT values were obtained for both methylated and unmethylated products. As shown in Table 3, there was a significant difference of the q‐MSP product type between NPC and control group. For both Cp and Wp detection, control samples were predominantly type U (>90.00%), while most NPC samples showed amplification of methylated products (type M or MU). Therefore, using the detection of methylated products as the diagnostic criterion for NPC, the amplification of methylated products was considered a positive result. As shown in Table 4, the specificity of the NPC diagnostic method was high for all four types of samples (>90.00%). For both Cp and Wp as detection markers, the sensitivity of the diagnostic method was highest in oropharyngeal swab samples (Cp 78.18% and Wp 79.66%), followed by saliva samples (Cp 60.71% and Wp 69.33%).

**TABLE 3:** The distribution of product types in four types of samples.

Group	Product type	Saliva	Oropharyngeal swab	Oral swab	Mouthwash
Cp					
NPC	M^a^	27/56 (48.21%)	33/55 (60.00%)	7/22 (31.82%)	12/25 (48.00%)
	U^b^	22/56 (39.29%)	12/55 (21.82%)	12/22 (54.55%)	13/25 (52.00%)
	MU^c^	7/56 (12.50%)	10/55 (18.18%)	3/22 (13.64%)	0/25 (0.00%)
Control	M^a^	0/89 (0.00%)	2/51 (3.92%)	1/30 (3.33%)	0/32 (0.00%)
	U^b^	87/89 (97.75%)	48/51 (94.12%)	29/30 (96.67%)	32/32 (100.00%)
	MU^c^	2/89 (2.25%)	1/51 (1.96%)	0/30 (0.00%)	0/32 (0.00%)
Wp					
NPC	M^a^	42/75 (56.00%)	34/59 (57.63%)	12/31 (38.71%)	15/30 (50.00%)
	U^b^	23/75 (30.67%)	12/59 (20.34%)	15/31 (48.39%)	13/30 (43.33%)
	MU^c^	10/75 (13.33%)	13/59 (22.03%)	4/31 (12.90%)	2/30 (6.67%)
Control	M^a^	1/98 (1.02%)	1/57 (1.75%)	0/35 (0.00%)	0/38 (0.00%)
	U^b^	92/98 (93.88%)	55/57 (96.49%)	35/35 (100.00%)	38/38 (100.00%)
	MU^c^	5/98 (5.10%)	1/57 (1.75%)	0/35 (0.00%)	0/38 (0.00%)

Abbreviations: Cp, C promoter; CTm, CT value of the methylated product; CTu, CT value of the unmethylated product; NPC, nasopharyngeal carcinoma; Wp, W promoter.

^a^ Only CTm values were obtained.

^b^ Only CTu values were obtained.

^c^ CTm and CTu were both obtained.

**TABLE 4:** The diagnostic performance of methylation markers C promoter (Cp) and W promoter (Wp) for cases in the four types of samples.

Sample type	Predict	NPC	Control	Sensitivity (%)	Specificity (%)	PPV (%)	NPV (%)
Cp							
Saliva	+	34	2	60.71	97.75	94.44	79.82
	–	22	87				
Oropharyngeal swab	+	43	3	78.18	94.12	93.48	80.00
	–	12	48				
Oral swab	+	10	1	45.45	96.67	90.91	70.73
	–	12	29				
Mouthwash	+	12	0	48.00	100.00	100.00	71.11
	–	13	32				
Wp							
Saliva	+	52	6	69.33	93.88	89.66	80.00
	–	23	92				
Oropharyngeal swab	+	47	2	79.66	96.49	95.92	82.09
	–	12	55				
Oral swab	+	16	0	51.61	100.00	100.00	70.00
	–	15	35				
Mouthwash	+	17	0	56.67	100.00	100.00	74.51
	–	13	38				

Abbreviations: NPC, nasopharyngeal carcinoma; NPV, negative predictive value; PPV, positive predictive value.

### 2.2 EBV DNA load and EBV DNA methylation was analyzed in paired saliva and oropharyngeal swab

**FIGURE 2:**
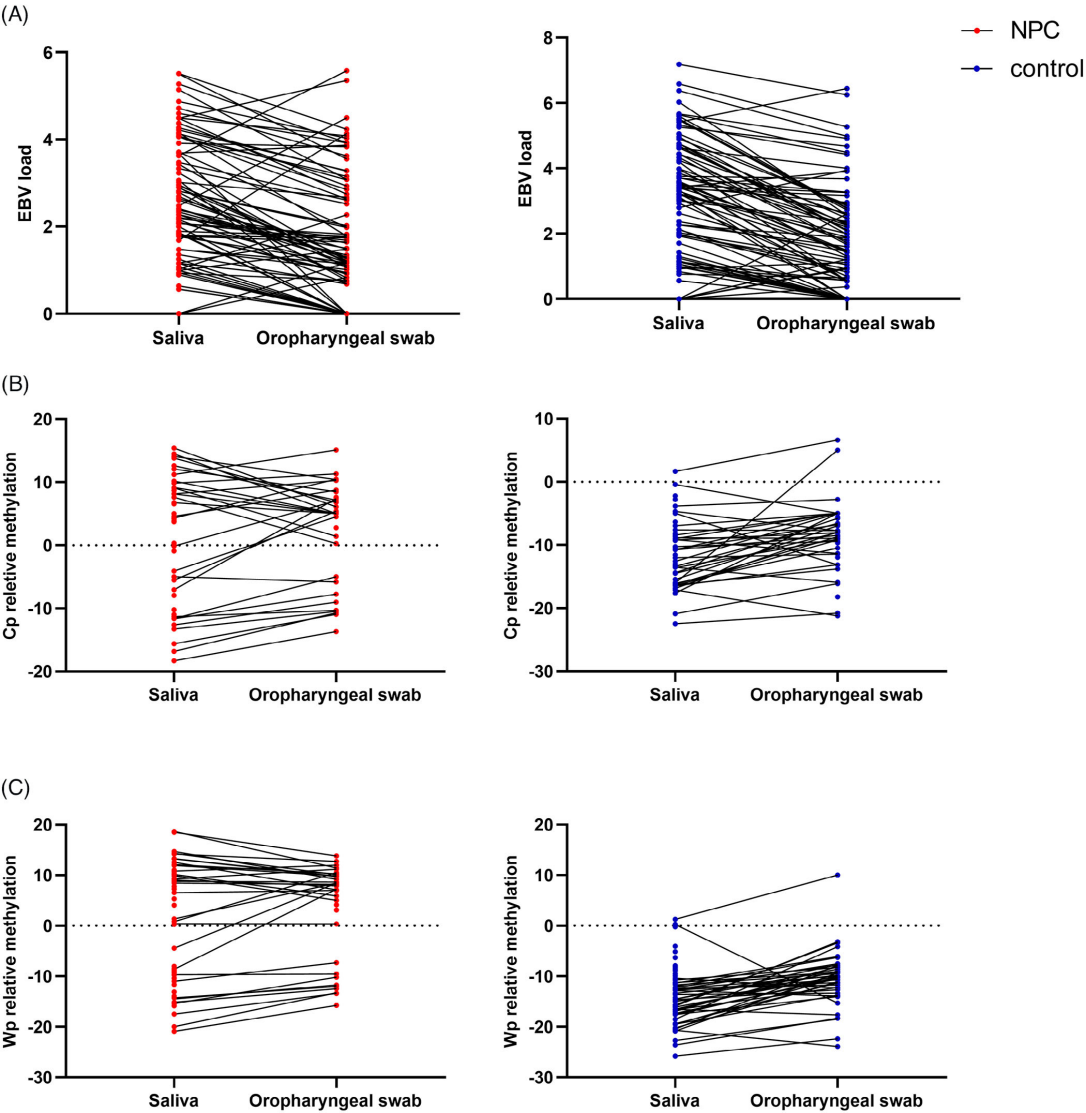
Epstein‒Barr virus (EBV) DNA load and EBV DNA methylation in paired saliva and oropharyngeal swab samples from nasopharyngeal carcinoma (NPC) patients and controls. (A) Comparison of EBV DNA load in paired saliva and oropharyngeal swab samples. The methylation levels of C promoter (Cp) region (B) and W promoter (Wp) region (C) in paired saliva and oropharyngeal swab samples.

Based on the above results, the detection rate and diagnostic performance were relatively better in oropharyngeal swab and saliva samples. Therefore, the following analysis further compared the detection in paired oropharyngeal swab and saliva samples. Among the samples, paired saliva and oropharyngeal swab were collected from 194 participants, including 89 NPC and 105 non‐NPC control. In detection of EBV DNA, saliva samples showed a higher EBV DNA detection rate and load (Table S3 and Figure 2A).

For EBV DNA methylation detection, saliva samples showed a higher detection rate in both CpG sites from Cp region (75.00%) and Wp region (81.41%). Oropharyngeal swab samples showed a lower detection rate (Cp 55.97% and Wp 61.94%). As for methylation levels, there was no difference in the relative methylation levels of both Cp and Wp between the two sampling methods for the NPC samples (*p* > 0.05). In the control samples, the relative methylation level was lower in saliva samples (*p* < 0.05). Nevertheless, both types of samples showed significant differences in methylation levels between groups, with the NPC group having higher methylation levels than the non‐NPC control group (Table S4 and Figure 2B,C). In addition, in the control group, the methylation levels in both types of samples were less than 0. In NPC detection, although the specificity was high for both types of samples (>90.00%), the sensitivity of the detection method was higher for oropharyngeal swab samples (Cp 73.53% vs. 64.15%; Wp 76.32% vs. 65.52%).

### 2.3 EBV DNA load and EBV DNA methylation was further analyzed in the different components of a saliva sample

Detection was further conducted for three different components of saliva samples. Fifty‐one saliva samples were divided into three groups: non‐centrifuged saliva (referred to as “saliva” in table and figure), centrifuged supernatant, and centrifuged precipitate.

As shown in Table S5, both EBV DNA detection rates and EBV DNA load of non‐centrifuged saliva and centrifuged precipitate samples were relatively higher compared to centrifuged supernatant samples. More than 90% of non‐centrifuged saliva and centrifuged precipitate samples were able to detect EBV DNA load, whereas only 84.31% was detected in centrifuged supernatant. Saliva and centrifuged precipitate samples also outperformed centrifuged supernatant samples in EBV DNA methylation detection. Taking the Cp detection as an example, the methylation detection rate of Cp in non‐centrifuged saliva, centrifuged precipitate, and centrifuged supernatant were 65.22%, 66.67%, and 41.86%, respectively. In both non‐centrifuged saliva and centrifuged precipitate samples, the intergroup differences in Cp and Wp methylation persisted, while Cp methylation differences were absent in the centrifuged supernatant samples (*p* = 0.174) (Table S6).

### 2.4 The combination of sampling types and detection sites could increase the detection of NPC

The above results indicated that saliva samples had a higher detection rate while oropharyngeal swab samples had a better diagnostic performance. In addition, although the methylation markers Cp and Wp had similar diagnostic performance in the detection of NPC, the detection for Wp had a higher detection rate. Therefore, we explored the complementarity of different sample types and different markers for NPC diagnosis within paired samples.

Different diagnostic methods were constructed based on combinations of sample types and methylation markers. The diagnostic criteria were as follows: successful detection was defined as the amplification of products being detected in any marker in any sample, while a positive diagnosis was determined if amplification of methylated products was observed in any marker in any sample. The detection rate and diagnostic performance of different diagnostic methods are shown in Table 5. Compared to other methods, combining two sampling methods and two markers for constructing a diagnostic method results in an increased detection rate (NPC 75.28% and control 68.57%), with a sensitivity of 74.63% and a specificity of 97.22%. The positive predictive value and negative predictive value were 96.15% and 80.46%, respectively.

**TABLE 5:** The detection rate and diagnostic performance of different diagnostic methods.

Diagnostic method^a^		Detection rate (%)		Diagnostic performance			
Sample type	Marker	NPC	Control	Sensitivity (%)	Specificity (%)	PPV (%)	NPV (%)
Saliva	Cp	53/89 (59.55)	64/105 (60.95)	64.15	96.88	94.44	76.54
Saliva	Wp	58/89 (65.17)	69/105 (65.71)	65.52	94.20	90.48	76.47
Oropharyngeal swab	Cp	34/89 (38.20)	41/105 (39.05)	73.53	95.12	92.59	81.25
Oropharyngeal swab	Wp	38/89 (42.70)	45/105 (42.86)	76.32	95.56	93.55	82.69
Saliva	Cp + Wp	63/89 (70.79)	71/105 (67.62)	66.67	94.37	91.30	76.14
Oropharyngeal swab	Cp + Wp	43/89 (48.31)	48/105 (45.71)	76.74	95.83	94.29	82.14
Saliva + oropharyngeal swab	Cp	57/89 (64.04)	70/105 (66.67)	70.18	95.71	93.02	79.76
Saliva + oropharyngeal swab	Wp	64/89 (71.91)	70/105 (66.67)	70.31	92.86	90.00	77.38
Saliva + oropharyngeal swab	Cp + Wp	67/89 (75.28)	72/105 (68.57)	74.63	97.22	96.15	80.46

Abbreviations: Cp, C promoter; NPC, nasopharyngeal carcinoma; NPV, negative predictive value; PPV, positive predictive value; Wp, W promoter.

^a^ A diagnosis was considered positive if methylated products were detected in either marker in any sample.

## 3 DISCUSSIONS

The convenience of sampling methods will greatly promotes the cancer detection. Saliva biopsy of NPC by detecting EBV DNA methylation were developed in our latest study,^25^ indicating the potential application of samples from oral cavity in the detection of NPC. In this study, we further investigated the effectiveness of EBV DNA methylation as tumor biomarkers for NPC detection among different self‐sampling methods from oral cavity, including saliva, oropharyngeal swab, oral swab, and mouthwash.

In fact, all four types of samples could be non‐invasively collected by donors themselves, and some of them have been employed for the detection and monitoring of certain diseases.^26^, ^27^ Therefore, self‐sampling methods allow for regular follow‐up monitoring at relatively low cost, facilitating early detection of signs of disease occurrence or recurrence, and thus aiding in optimizing the long‐term outcomes of NPC. However, each sample type also has some disadvantages. For example, collecting pure saliva samples in a non‐feeding state takes a relatively long time. Furthermore, the occurrence of dry mouth symptoms in some patients following treatment could result in difficulties in obtaining saliva samples. In contrast, mouthwash sample allows for quick sample collection within a short time by a rapid oral rinse, making it more convenient. However, it seems to dilute the saliva and introduce some other oral components. Compared to the aforementioned sampling methods, oropharyngeal swab and oral swab sampling are also convenient and rapid, and the sample component is purer. However, these two sampling methods also exhibit shortcomings, such as there is some subjectivity of this sampling process. Therefore, further detection of EBV DNA load and EBV DNA methylation in these samples is worth exploring in order to determine the best sampling method and diagnostic performance.

In terms of EBV DNA load, saliva samples showed the highest detection rate and detection quantity (Table 1), followed by oropharyngeal swab and oral swab samples, while mouthwash samples exhibited the lowest performance. It is widely regarded EBV in the oral cavity is primarily released from lymphocytes in the Waldeyer's ring, and amplification of lytic replication in surrounding epithelial cells occurs.^28^, ^29^ Finally, free EBV virion is continuously released into the saliva so that they can spread from person to person. Unlike oropharyngeal mucosal, buccal mucosa is a little far away from the Waldeyer's ring, while the mouthwash sample appears to be diluted. Therefore, performance of oral swab and mouthwash was inferior to that of the other two sample types. However, it seemed there was a higher EBV DNA load and detection rate in saliva and oropharyngeal swab. The results suggested that these two sampling methods could be firstly recommended for future applications.

In this study, the previously identified 11,029 CpG site within the single‐copy Cp region and the newly proposed 12,420 CpG site within the repeat‐copy Wp region were detected. As expected, the results showed the detection of Wp region had an increase compared to the detection rate of Cp region (Table 2), especially because of its advantage when detecting samples with low EBV DNA load. Therefore, it seemed detection of CpG site within Wp region would be more useful in the detection of NPC. Despite the presence of some difference in detection rates, EBV DNA methylation detection, including methylation level and methylation type, among different sample types were highly consistent. First, the methylation level or methylation degree almost all had a significant increase in all the four sample types from NPC patients, no matter in Wp detection or in Cp detection (Table 2 and Figure 1). Not only that, the methylation level of the CpG site was found to decrease below the COV in NPC patients after therapy, and increase after recurrence in our previous study.^22^ In this study, the methylation level of the CpG site in a dynamic collection of salivary samples from 10 out of 30 healthy individuals was further detected, and it was consistently stable in these samples (Figure S1). Second, there was also a significant difference in the methylation type between NPC patients and controls (Table 3). In order to investigate whether there was the EBV DNA methylation modifications in the oral cavity in patients with other tumors, this study simultaneously recruited some patients such as lymphoma and other head and neck squamous cell carcinoma (HNSCC). It should be noted that EBV was predominantly in a non‐methylated state not only in non‐tumor controls but also in other tumor controls, even those with some EBV‐related lymphoma (Figure S2). It seemed that the changes in EBV DNA methylation modifications in NPC were unique and may be related to the mechanism by which the EBV contributes to the development of NPC.

As observed in our previous study, there were no CT values for methylated DNA templates in vast majority of samples from controls, indicating that the EBV DNA was unmethylated. However, CT values for methylated DNA templates were detected in majority of samples from NPC patients, even though only CT value could be detected, indicating that there was almost no unmethylated EBV DNA in the samples (Table 3). It is widely considered that DNA of free EBV virion from lytic replication is unmethylated and free of any epigenetic modification.^30^ When entering host cells, EBV genome becomes hypermethylated, regulating a number of viral transcripts.^3^ Combined with the observations from this study, we propose the following hypothesis: in healthy people, EBV was continuously released into oral samples such as saliva and was transmitted from person to person.^31^ Therefore, unmethylated EBV DNA could be detected. When NPC occurs, EBV lytic infection was suppressed and latent infection was predominant. This shift was consistent across the entire oral cavity, but the extent in different regions of mouth may not be completely the same. Because the proportion of only non‐methylated products (U) amplified was relatively higher in oral swab and mouthwash samples compared to oropharyngeal swab and saliva samples from NPC patients. It was speculated that this transition within NPC predominantly or first occurred in the pharynx.

The methylated EBV DNA may come from cells with EBV latent infection, or come from cell lysis release, or both of them. Similarly, unmethylated EBV DNA may come from cells with EBV lytic infection, or come from free EBV virion, or both of them. In order to further investigate the infection status of EBV in the oral cavity, EBV DNA from different components, including non‐centrifuged saliva, centrifuged supernatant, and centrifuged precipitate, was further detected. Although the detection rate of EBV DNA load and EBV DNA methylation had a decrease in the supernatant (Table S5), the methylation level and methylation type in all components were basically consistent (Tables S6 and S7). It was concluded that the methylated EBV DNA detected in NPC samples was not only from intact cells, but also partially released from ruptured cells. The unmethylated EBV DNA in control samples was from intact viral particles that had been released extracellularly or were present within cells but had not yet been released. This result further corroborates the aforementioned hypothesis: during the carcinogenic process of NPC, the lytic release of EBV was blocked and mainly in a latent infection. However, further experiments are required for comprehensive validation and elucidation of this phenomenon.

The findings of this study showed that saliva and oropharyngeal swabs had a relatively better detection rates and diagnostic performance for EBV DNA methylation. In our previous study involving a dynamic collection of salivary samples from a healthy population, we discovered that the EBV DNA load within saliva undergoes dynamic fluctuations.^22^ Moreover, each individual displayed at least one sample with detectable levels of EBV during the 3‐month consecutive sampling period. This result indicated that there was a certain degree of randomness in a single sampling. In this study, both types of sampling were performed at the same time to explore whether detection efficiency could be improved. As expected, the combined utilization of saliva and oropharyngeal swab sampling methods, along with the assessment of Cp and Wp methylation indicators, demonstrated a certain level of enhancement in detection rates while maintaining diagnostic efficacy (Table 5). This indicated that the joint sampling of different oral regions can complement single‐sampling methods, potentially resulting in improved results. Theoretically, this parallel approach is more practical and acceptable, although it needs to be validated through further studies with a larger sample size.

There were also some limitations in this study. For example, current results suggested that EBV‐infected cells and nucleic acid fragment might contribute to the methylated EBV DNA in NPC patients. However, whether this DNA came from NPC tumor cells or tumor cell lysis was not clear. Cancer cells from nasopharynx into saliva were easy to understand. However, this could not explain the detection of methylated EBV DNA in oral swabs. Further experiments are needed in the future. Besides, bisulfate treatment would cause the DNA degradation, leading to the detection failure of EBV DNA methylation. Therefore, it is worth to explore other methods to improve the detection rate. In fact, isolating the methylated EBV DNA from total DNA by methyl CpG binding magnetic beads have been carried out by our group to improve this problem. The methylation level can be reflected by calculating the EBV DNA load in the sample after and before isolation. More importantly, by combining with the microfluidic technology developed by us, point of care testing of EBV DNA methylation may be possible. Third, it was hard to evaluate its performance in early‐stage patients because EBV DNA methylation in saliva and oropharyngeal swab was only successfully detected in two samples from early‐stage patients in this cohort. Although seven out of 12 saliva samples from NPC patients in stage I/II disease were observed to have elevated methylation level in our previous study, more samples will be needed for evaluation in the future.

In conclusion, our study explored the performance of four different oral sampling methods for detecting NPC using EBV DNA methylation as a biomarker. Saliva and oropharyngeal swab showed the best performance, while oral swab and mouthwash were relatively less effective. The combination of sampling type and detection sites could increase the detection of NPC, potentially advancing its application in non‐clinical screening for NPC.

## 4 MATERIALS AND METHODS

### 4.1 Patients and controls

A total of 495 participants were newly recruited for this study, including 252 non‐NPC controls and 243 NPC. Control subjects contained non‐tumor controls (*n* = 156) and other tumor controls (*n* = 96). NPC and non‐tumor control samples were recruited from the nasopharyngology outpatient department. Samples with positive pathology results were classified as NPC, while those with negative results were classified as non‐tumor controls. The other tumor controls included subjects with lymphoma, other HNSCC, and other types of tumors such as melanoma, colorectal cancer, and esophageal cancer. From these participants, 907 various samples from oral cavity, including 262 saliva, 250 oropharyngeal, 210 oral swab, and 185 mouthwash samples, were collected. Of these, 194 pairs were paired samples, and both saliva and oropharyngeal swabs were collected. Furthermore, different components of 51 out of these 54 saliva samples, including non‐centrifuged saliva, centrifuged supernatant, and centrifuged precipitate, were also obtained. All participants were recruited at Sun Yat‐Sen University Cancer Center (Guangzhou, China). The demographic data for subjects evaluated in this study are summarized in Table S8.

### 4.2 Saliva collection

All saliva samples were collected and processed by standard procedures as reported in our previous studies.^32^, ^33^ Briefly, subjects were not advised to eat or drink before sample collection for at least 30 min. In this study, 2–3 mL pure saliva samples were collected, aliquoted, and finally frozen at ‒80°C before DNA extraction.

### 4.3 Oropharyngeal and oral swab collection

In theory, oropharyngeal and oral swab can also be collected by donors themselves. However, sampling was currently performed by a nurse in this study. Prior to biopsy, two swabs (Copan Diagnostics) were inserted into the oral cavity in sequence. The first swab, defined as the oral swab, was rotated several times over the buccal sides of the mouth and then removed. The second swab, defined as the oropharyngeal swab, was inserted into the oropharynx, rotated several times over the oropharyngeal epithelium, and removed quickly. Immediately after sampling, the swab head (1.5 cm) was cut off and placed in 400 μL of phosphate‐buffered saline (PBS; Cytiva) and finally stored at −80°C until DNA extraction.

### 4.4 Mouthwash collection

All mouthwash samples were collected and processed by standard procedures as reported in our previous studies.^32^, ^33^ Briefly, 30 min before sampling, the patients were advised not to eat or drink anything. Then, each patient was instructed to rinse their mouth with 5 mL of physiological saline (0.9% NaCl) for 30 s. The mouthwash was aliquoted and frozen at ‒80°C before DNA extraction.

### 4.5 DNA extraction

The total DNA was extracted using an automated workstation (Hamilton Robotic, Bonaduz, GR). Based on the aforementioned sample collection methods, the original volumes of each type of sample were as follows: saliva (2‒3 mL), oral swab (400 μL), oropharynx swab (400 μL), and mouthwash (5 mL). Among them, the uncentrifuged saliva referred to the saliva samples that had not undergone centrifugation. The centrifuged saliva samples undergone centrifugation at 1000 RPM for 10 min to separate into precipitate and supernatant. In the precipitate samples, 400 μL of PBS was added for dissolution. Before DNA extraction, each type of sample was thoroughly mixed, and 400 μL of the original sample solution was used for DNA extraction. Following the protocol of the automated workstation, sequentially performed lysis, magnetic bead binding, and elution operations were conducted on the samples, and finally 100 μL of DNA sample was obtained.

### 4.6 EBV DNA load quantification by quantitative real‐time PCR

The EBV DNA loads in samples were analyzed by quantitative real‐time PCR (q‐PCR), as described previously.^16^, ^21^ Samples with CT values were considered to have a positive EBV DNA load; in contrast, they were considered as a negative sample. Detection rate was defined as the proportion of positive samples to the total samples. The EBV DNA load in samples was expressed as copies/μL. During the analysis, the EBV DNA loads were transformed as follows: log_10_(copies/μL + 1).

### 4.7 EBV DNA methylation quantification by qMSP

First, extracted sample DNA was bisulfite transformed using the EZ DNA Methylation‐Gold kit (Zymo Research). The maximum conversion volume (20 μL) was used for DNA bisulfite conversion. Fully methylated and fully unmethylated probes containing the target sites (11,029 bp in Cp region and 12,420 bp in Wp region) were used for relative quantitative detection of methylation. PCR reaction was consistent with previous descriptions.^22^, ^25^ CT values could be obtained for both methylated (CTm) and unmethylated (CTu) products. If CTm or CTu had a detectable value, it was considered as a positive result for EBV DNA methylation detection; otherwise, it was considered as a failed sample for EBV DNA methylation detection. For samples that tested positive but lacked a CTm or CTu value, CT = 45 was used for the subsequent calculations. The detection rate was defined as the proportion of positive samples to the total samples. The methylation level was calculated by determining the ΔCT values: (CTm ‒ CTu). When ΔCT was greater than 0, it suggested that the sample primarily contained methylated EBV DNA. Conversely, when ΔCT was less than 0, it suggested the prevalence of unmethylated EBV DNA. The methylation type was qualitatively analyzed based on whether CT values were detected. Quality assessment for the methylation detection was conducted using two standard plasmids, each containing either the completely methylated or the completely unmethylated sequence. According to the result, the amplification efficiencies of Wp‐methylated and Wp‐unmethylated products were 92.03% and 91.28%, respectively. The amplification efficiencies of Cp‐methylated and Cp‐unmethylated products were 106.66% and 101.19%, respectively (Table S9). The amplification efficiency met the standard requirements (90.00%‒110.00%). All primer and probe sequences used in this research are listed in Table S10.

### 4.8 Statistical analyses

The Wilcoxon rank sum test was utilized to analyze the variations in EBV DNA load and EBV DNA methylation levels between the NPC and control groups for all sample types. The *χ* ^2^‐test and Fisher's exact probability test were used to assess the differences in detection rates among different sample types. The Kruskal‒Wallis test was used to test for differences in EBV DNA load and the methylation level among different sample types. A *p*‐value of less than 0.05 was considered statistically significant for an analysis. All statistical analyses were conducted using R (4.2.1) software.

## AUTHOR CONTRIBUTIONS

X.H.Z. and W.H.J. conceived and designed the study. X.H.Z., X.Z.L., and C.L.T. carried out the experiments, performed data collection, and drafted the manuscript. Y.M.Z., T.Z., and X.J.Y. assisted the experiments and made contributions to statistical analyses. Y.L., Y.Q.H., T.M.W., and W.Q.X. recruited the participants. X.H.Z. and C.L.T. completed the manuscript with intellectual input from W.H.J. The work reported in the paper has been performed by the authors, unless clearly specified in the text. All authors approved the final manuscript.

## CONFLICT OF INTEREST STATEMENT

The authors declare they have no conflicts of interest.

## ETHICS STATEMENT

This study was approved by the Institutional Review Board of the Sun Yat‐Sen University Cancer Center, and all participants provided informed consents (ethical approval number: B2022‐321‐01).

## Supporting information

Supporting Information

## Data Availability

The datasets used and analyzed during the current study are available from the corresponding author on reasonable request.
