# Supplementary material for: Detection of Epstein‒Barr virus DNA methylation as tumor markers of nasopharyngeal carcinoma patients in saliva, oropharyngeal swab, oral swab, and mouthwash
Source: MedComm (2020). 2024 Aug 19;5(9):e673. doi: 10.1002/mco2.673 (PMC11331033; doi:10.1002/mco2.673)
Supplement: Supplementary file 1 — Supporting Information [file MCO2-5-e673-s001.doc]

Detection of Epstein Barr virus DNA methylation as tumor markers of nasopharyngeal carcinoma patients in saliva, oropharyngeal swab, oral swab and mouthwash

Xiao-Hui Zheng^1#^*, Xi-Zhao Li^1#^, Cao-Li Tang^2#^, Yu-Meng Zhang^2^, Ting Zhou^1^, Xiao-Jing Yang^1^, Ying Liao^1^, Yong-Qiao He^1^, Tong-Min Wang^1^, Wen-Qiong Xue^1^ and Wei-Hua Jia^1,2^*

[1] State Key Laboratory of Oncology in South China, Guangdong Key Laboratory of Nasopharyngeal Carcinoma Diagnosis and Therapy, Guangdong Provincial Clinical Research Center for Cancer, Sun Yat-sen University Cancer Center, Guangzhou 510060, China

[2] School of Public Health, Sun Yat-sen University, Guangzhou 510080, China

[*] Corresponding Author:

Prof. Xiao-Hui Zheng

Sun Yat-sen University Cancer Center

651 Dongfeng East Road, Guangzhou, Guangdong 510060, China

Tel: 8620 8734 3370; Fax: 8620 8734 3392

Email: [zhengxh@sysucc.org.cn](mailto:jiawh@sysucc.org.cn)

Prof. Wei-Hua Jia

Sun Yat-Sen University Cancer Center

651 Dongfeng East Road, Guangzhou, Guangdong 510060, P. R. China

Tel: 8620 8734 2327; Fax: 8620 8734 3392

Email: [jiawh@sysucc.org.cn](mailto:jiawh@sysucc.org.cn)

[#] These authors contributed equally to this work.


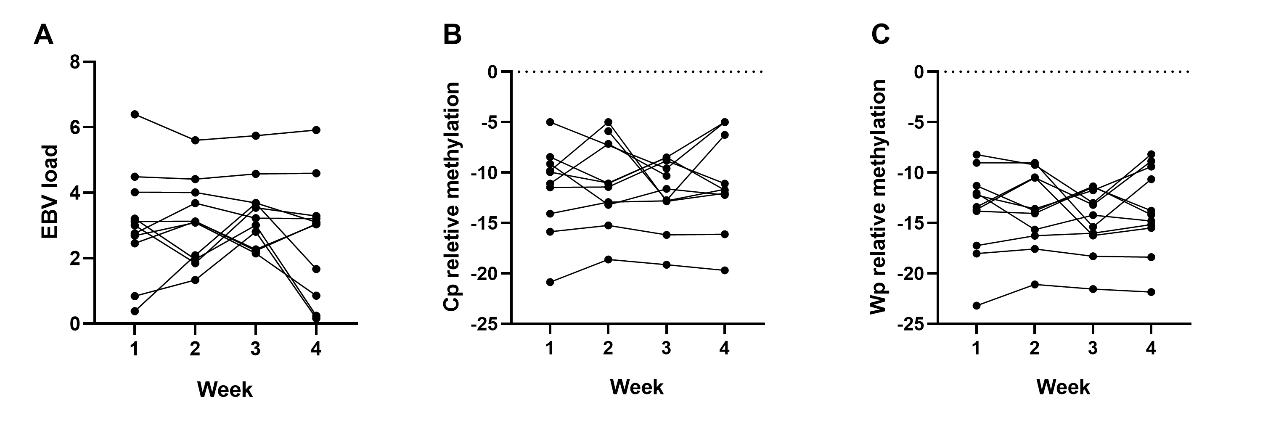


**Figure S1. EBV detection in saliva samples collected from healthy donors at four consecutive one-week intervals.** **A.** EBV load in saliva samples collected from healthy donors at four consecutive one-week intervals. **B.** The methylation of Cp in saliva samples. **C.** The methylation of Wp in saliva samples.


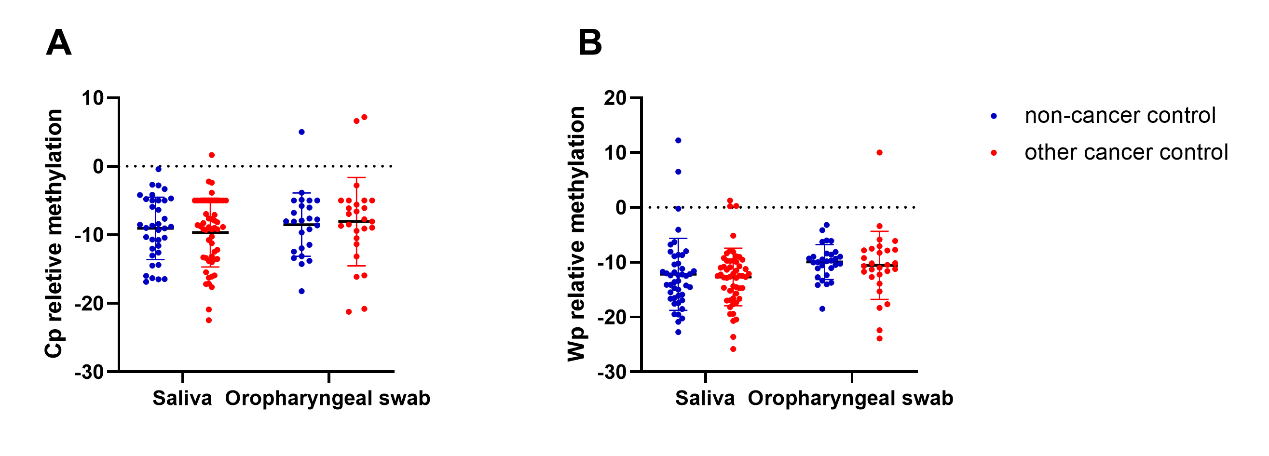


**Figure S2. Comparison of EBV methylation levels in different control samples. A.** The Cp methylation levels in different control samples. **B.** The Wp methylation levels in different control samples.

| **Table S1.** Comparison of EBV load and methylation in samples of different ages. | | | | | | | | | | | |
| --- | --- | --- | --- | --- | --- | --- | --- | --- | --- | --- | --- |
|  | NPC | | | | |  | control | | | | |
| Age | <40 | 40-49 | 50-59 | >=60 | *P* |  | <40 | 40-49 | 50-59 | >=60 | *P* |
| Saliva | | | | | | | | | | | |
| N | 27 | 37 | 32 | 22 |  |  | 40 | 39 | 42 | 23 |  |
| EBV load | 1.41(0.16,2.12) | 2.18(0.99,2.89) | 2.68(1.12,3.69) | 2.41(1.16,3.87) | 0.0541 |  | 2.04(0.00,3.60) | 2.17(0.28,3.48) | 2.32(0.00,3.85) | 3.30(0.00,4.65) | 0.5680 |
| Cp | 4.43(-4.32,7.93) | 2.27(-6.31,5.44) | 4.32(-4.77,8.16) | -5.00(-11.32,6.75) | 0.4667 |  | -8.77(-11.70,-5.00) | -8.41(-9.29,-5.00) | -9.15(-13.59,-6.96) | -11.24(-16.18,-6.57) | 0.1162 |
| Wp | 6.57(-8.62,8.45) | 6.55(-2.03,9.12) | 7.83(-4.05,11.78) | 5.26(-12.63,9.43) | 0.5554 |  | -12.39(-15.00,-10.76) | -12.23(-14.77,-9.55) | -12.76(-17.34,-10.38) | -14.54(-17.00,-9.94) | 0.8660 |
| Oral swab | | | | | | | | | | | |
| N | 24 | 42 | 30 | 23 |  |  | 24 | 23 | 26 | 18 |  |
| EBV load | 0.47(0.00,1.08) | 0.72(0.00,1.21) | 1.02(0.58,1.93) | 1.55(0.39,2.25) | 0.0259 |  | 0.41(0.00,1.44) | 1.25(0.19,1.74) | 0.89(0.00,1.88) | 1.66(0.09,2.55) | 0.3415 |
| Cp | -3.23(-5.62,0.88) | 4.36(-6.90,4.79) | -5.00(-5.00,3.53) | -5.12(-10.35,5.59) | 0.8421 |  | -9.30(-11.12,-8.66) | -9.32(-12.49,-6.14) | -10.84(-12.53,-6.51) | -10.21(-14.76,-4.58) | 0.9064 |
| Wp | -6.38(-8.20,-1.76) | -4.05(-11.01,3.72) | 5.03(-0.00,8.56) | -8.02(-12.47,3.22) | 0.1705 |  | -9.37(-10.52,-8.65) | -8.70(-11.24,-7.46) | -10.70(-13.67,-9.16) | -9.62(-10.68,-7.65) | 0.7758 |
| Oropharyngeal swab | | | | | | | | | | | |
| N | 24 | 42 | 30 | 23 |  |  | 38 | 32 | 36 | 25 |  |
| EBV load | 1.57(0.52,2.86) | 1.29(0.17,2.54) | 1.98(1.20,3.57) | 1.78(0.89,2.95) | 0.1437 |  | 0.60(0.00,1.92) | 0.96(0.00,1.70) | 1.28(0.00,2.62) | 2.09(0.56,3.11) | 0.0427 |
| Cp | 4.98(2.97,6.62) | 5.00(2.20,5.65) | 5.71(3.91,9.91) | -1.79(-9.34,4.61) | 0.1124 |  | -8.68(-9.54,-7.14) | -8.50(-12.68,-5.83) | -6.95(-8.05,-5.57) | -7.74(-13.70,-5.00) | 0.7022 |
| Wp | 8.02(5.00,9.54) | 7.24(4.81,8.54) | 9.16(3.32,10.51) | 2.62(-11.10,9.52) | 0.2336 |  | -10.03(-12.02,-8.79) | -8.98(-11.73,-6.10) | -10.38(-11.09,-9.61) | -9.70(-14.68,-7.80) | 0.5007 |
| Mouthwash | | | | | | | | | | | |
| N | 15 | 21 | 38 | 23 |  |  | 24 | 37 | 22 | 5 |  |
| EBV load | 1.38(0.00,2.63) | 0.59(0.00,1.59) | 0.00(0.00,1.23) | 1.86(0.00,3.08) | 0.0516 |  | 1.73(0.00,2.62) | 1.29(0.00,2.37) | 1.38(0.00,2.28) | 0.00(0.00,1.51) | 0.6043 |
| Cp | 5.35(-6.11,8.54) | -1.00(-4.28,2.27) | 3.73(-8.47,6.77) | -6.59(-10.31,4.37) | 0.5870 |  | -7.15(-8.95,-5.51) | -5.74(-9.45,-4.14) | -5.15(-6.17,-4.20) | NA | 0.8114 |
| Wp | 4.33(-0.56,6.41) | 7.15(-2.50,7.54) | -0.38(-11.45,5.89) | -8.24(-12.12,6.37) | 0.5267 |  | -8.36(-11.03,-6.32) | -8.52(-11.37,-7.28) | -8.16(-10.77,-7.33) | -6.05(-6.05,-6.05) | 0.8536 |
| NA: No samples were detected. | | | | | | | | | | | |

| **Table S2.** Comparison of EBV load and methylation in samples of different genders. | | | | | | | |
| --- | --- | --- | --- | --- | --- | --- | --- |
|  | NPC | | |  | control | | |
| Sex | Male | Female | *P* |  | Male | Female | *P* |
| Saliva | | | | | | | |
| N | 83 | 35 |  |  | 82 | 62 |  |
| EBV load | 2.10(1.03,3.35) | 1.41(0.82,2.67) | 0.0524 |  | 2.57(0.99,3.60) | 1.96(0.00,4.20) | 0.3211 |
| Cp | 4.03(-5.00,8.56) | -0.20(-5.95,6.16) | 0.6509 |  | -8.85(-12.09,-5.00) | -9.03(-13.48,-5.00) | 0.4403 |
| Wp | 8.31(-8.96,10.57) | 3.90(-9.02,7.62) | 0.5394 |  | -12.37(-14.71,-10.38) | -14.22(-16.56,-8.88) | 0.6077 |
| Oral swab | | | | | | | |
| N | 86 | 33 |  |  | 43 | 48 |  |
| EBV load | 0.92(0.00,1.74) | 0.83(0.00,1.07) | 0.3963 |  | 1.13(0.00,1.80) | 0.97(0.00,1.88) | 0.9583 |
| Cp | -4.12(-8.11,4.69) | -0.06(-6.28,5.00) | 0.6629 |  | -10.21(-12.53,-6.51) | -9.30(-12.67,-5.00) | 0.5942 |
| Wp | 2.78(-10.69,5.52) | -8.02(-11.36,-1.20) | 0.2628 |  | -9.63(-12.27,-8.40) | -9.05(-12.25,-7.66) | 0.7076 |
| Oropharyngeal swab | | | | | | | |
| N | 86 | 33 |  |  | 66 | 65 |  |
| EBV load | 1.71(0.92,3.08) | 1.48(0.74,2.83) | 0.6598 |  | 1.27(0.00,2.70) | 0.96(0.00,2.26) | 0.5777 |
| Cp | 5.00(1.08,7.07) | 4.53(1.52,6.89) | 0.5182 |  | -7.97(-9.05,-5.86) | -8.63(-12.45,-5.00) | 0.7491 |
| Wp | 7.24(2.69,9.83) | 8.02(6.08,9.99) | 0.5079 |  | -10.48(-11.67,-7.98) | -10.06(-13.48,-8.69) | 0.9666 |
| Mouthwash | | | | | | | |
| N | 70 | 27 |  |  | 57 | 31 |  |
| EBV load | 0.98(0.00,2.22) | 0.00(0.00,2.08) | 0.8217 |  | 1.65(0.00,2.49) | 0.00(0.00,1.97) | 0.1729 |
| Cp | -0.82(-7.93,5.33) | -6.11(-12.35,6.80) | 0.7038 |  | -5.15(-8.49,-4.16) | -6.43(-13.04,-5.84) | 0.1986 |
| Wp | 3.89(-9.69,6.37) | -8.77(-14.24,8.99) | 0.6661 |  | -8.15(-10.87,-6.78) | -9.14(-13.24,-8.53) | 0.4338 |

| **Table S3.** EBV DNA load in paired saliva and oropharyngeal swab samples. | | | |
| --- | --- | --- | --- |
|  | Saliva | Oropharyngeal swab | *P* |
| EBV detection rate^a^ (%) | | | |
| NPC | 80/89(89.89) | 67/89(75.28) | 0.0102 |
| control | 76/105(72.38) | 67/105(63.81) | 0.1827 |
| All | 156/194(80.41) | 134/194(69.07) | 0.0102 |
| EBV load [Median(P_25_,P_75_)] | | | |
| NPC | 2.28(1.25,3.63) | 1.29(0.68,2.63) | <0.001 |
| control | 2.37(0.00,3.98) | 1.05(0.00,2.38) | <0.001 |
| *P* | 0.8842 | 0.4242 |  |
| ^a^ Samples with EBV load>0 were considered positive | | |  |

| **Table S4.** EBV DNA methylation in paired saliva and oropharyngeal swab samples. | | | |
| --- | --- | --- | --- |
|  | Saliva | Oropharyngeal swab | *P* |
| Cp | | | |
| Detection rate (%) | | | |
| NPC | 53/80(66.25) | 34/67(50.75) | 0.0568 |
| control | 64/76(84.21) | 41/67(61.19) | 0.0019 |
| All | 117/156(75.00) | 75/134(55.97) | <0.001 |
| Relative methylation level [Median(P_25_,P_75_)] | | | |
| NPC | 4.19(-5.00,8.16) | 5.00(-3.68,7.32) | 0.4930 |
| control | -9.02(-13.43,-5.00) | -8.05(-11.34,-5.00) | <0.001 |
| *P* | <0.001 | <0.001 |  |
| Diagnostic performance | | | |
| Sensitivity (%) | 64.15 | 73.53 |  |
| Specificity (%) | 96.88 | 95.12 |  |
| PPV (%) | 94.44 | 92.59 |  |
| NPV (%) | 76.54 | 81.25 |  |
| Wp | | | |
| Detection rate (%) | | | |
| NPC | 58/80(72.50) | 38/67(56.72) | 0.0453 |
| control | 69/76(90.79) | 45/67(67.16) | <0.001 |
| All | 127/156(81.41) | 83/134(61.94) | <0.001 |
| Relative methylation level [Median(P_25_,P_75_)] | | | |
| NPC | 6.54(-9.59,10.23) | 8.03(1.06,9.80) | 0.3102 |
| control | -12.70(-16.65,-10.38) | -10.20(-12.37,-8.29) | <0.001 |
| *P* | <0.001 | <0.001 |  |
| Diagnostic performance | | | |
| Sensitivity (%) | 65.52 | 76.32 |  |
| Specificity (%) | 94.20 | 95.56 |  |
| PPV (%) | 90.48 | 93.55 |  |
| NPV (%) | 76.47 | 82.69 |  |

| **Table S5.** EBV DNA load in different components of a saliva sample. | | | | |
| --- | --- | --- | --- | --- |
|  | Supernatant | Precipitate | Saliva | *P* |
| EBV detection rate^a^ | |  |  |  |
| NPC | 28/31(90.32%) | 30/31(96.77%) | 30/31(96.77%) | 0.613 |
| control | 15/20(75.00%) | 18/20(90.00%) | 16/20(80.00%) | 0.589 |
| All | 43/51(84.31%) | 48/51(94.12%) | 46/51(90.20%) | 0.305 |
| EBV load^b^ [Median (P_25_, P_75_)] | |  |  |  |
| NPC | 1.48 (0.99, 2.53) | 2.49 (1.79, 3.66) | 2.48 (1.74, 3.66) | 0.018 |
| control | 2.05 (0.63, 3.41) | 3.39 (1.30, 4.24) | 3.51 (1.06, 4.70) | 0.268 |
| All | 1.59 (0.85, 3.09) | 2.78 (1.55, 3.98) | 2.69 (1.52, 4.21) | 0.011 |
| ^a^ Samples with EBV load>0 were considered positive; | | | | |
| ^b^The calculation method for EBV load was log10 (copy/μl+1). | | | | |

| **Table S6.** EBV DNA methylation in different components of a saliva sample. | | | | |
| --- | --- | --- | --- | --- |
|  | Supernatant | Precipitate | Saliva | *P*^a^ |
| Wp | | | | |
| Detection rate | |  |  |  |
| NPC | 9/28(32.14%) | 16/30(53.33%) | 16/30(53.33%) | 0.141 |
| control | 13/15(86.67%) | 12/18(66.67%) | 14/16(87.50%) | 0.301 |
| All | 22/43(51.16%) | 28/48(58.33%) | 30/46(65.22%) | 0.405 |
| Methylation level [Median (P_25_, P_75_)] | |  |  |  |
| NPC | 5.24 (-5.30, 8.57) | 7.67 (5.36, 12.21) | 9.13 (5.00, 11.26) | 0.426 |
| control | -11.37 (-14.41, -8.67) | -13.29 (-15.39, -11.77) | -14.37 (-16.66, -11.71) | 0.372 |
| *P*^b^ | 0.005 | <0.001 | <0.001 | - |
| Cp | | | | |
| Detection rate | |  |  |  |
| NPC | 12/28(42.86%) | 23/30(76.67%) | 20/30(66.67%) | 0.025 |
| control | 6/15(40.00%) | 9/18(50.00%) | 10/16(62.50%) | 0.294 |
| All | 18/43(41.86%) | 32/48(66.67%) | 30/46(65.22%) | 0.029 |
| Methylation level [Median (P_25_, P_75_)] | |  |  |  |
| NPC | -4.03 (-9.23, 7.84) | 6.02 (2.06, 9.74) | 4.45 (-0.33, 10.19) | 0.229 |
| control | -8.89 (-10.57, -5.77) | -10.02 (-13.38, -9.12) | -9.25 (-10.73, -7.01) | 0.83 |
| *P*^b^ | 0.174 | <0.001 | <0.001 | - |
| ^a^ P values for statistical tests among different sample types; ^b^ P values for statistical tests between NPC and control groups. | | | | |

| **Table S7.** The distribution of methylated types in different saliva components. | | | | |
| --- | --- | --- | --- | --- |
| Group | Type | Supernatant | Precipitate | Saliva |
| Wp | | | | |
| NPC | M^a^ | 5/9(55.56%) | 12/16(75.00%) | 12/16(75.00%) |
|  | M>U^b^ | 1/9(11.11%) | 1/16(6.25%) | 1/16(6.25%) |
|  | U^c^ | 3/9(33.33%) | 2/16(12.50%) | 2/16(12.50%) |
|  | U>M^d^ | 0/9(0.00%) | 1/16(6.25%) | 1/16(6.25%) |
| Control | M^a^ | 0/13(0.00%) | 0/12(0.00%) | 0/14(0.00%) |
|  | M>U^b^ | 0/13(0.00%) | 0/12(0.00%) | 0/14(0.00%) |
|  | U^c^ | 13/13(100.00%) | 11/12(91.67%) | 14/14(100.00%) |
|  | U>M^d^ | 0/13(0.00%) | 1/12(8.33%) | 0/14(0.00%) |
| Cp | | | | |
| NPC | M^a^ | 5/12(41.67%) | 15/23(65.22%) | 12/20(60.00%) |
|  | M>U^b^ | 0/12(0.00%) | 3/23(13.04%) | 2/20(10.00%) |
|  | U^c^ | 6/12(50.00%) | 3/23(13.04%) | 3/20(15.00%) |
|  | U>M^d^ | 1/12(8.33%) | 2/23(8.70%) | 3/20(15.00%) |
| Control | M^a^ | 0/6(0.00%) | 0/9(0.00%) | 0/10(0.00%) |
|  | M>U^b^ | 0/6(0.00%) | 0/9(0.00%) | 0/10(0.00%) |
|  | U^c^ | 6/6(100.00%) | 9/9(100.00%) | 10/10(100.00%) |
|  | U>M^d^ | 0/6(0.00%) | 0/9(0.00%) | 0/10(0.00%) |
| ^a^Only CTm values were obtained;  ^b^CTm and CTu was both obtained, but the value of CTm was less than its value of CTu;  ^c^Only CTu values were obtained;  ^d^CTm and CTu was both obtained, but the value of CTu was less than its value of CTm | | | | |

| **Table S8.** The demographic characteristics of the population from which the samples were derived. | | | | | | | | |  |
| --- | --- | --- | --- | --- | --- | --- | --- | --- | --- |
|  | Control | | | | | | NPC | *P*^a^ |  |
|  | Non-cancer control | Other-cancer control | | | | All |  |  |  |
|  |  | Lymphoma | HNSCC^b^ | Other | All |  |  |  |  |
| N | 156 | 34 | 30 | 32 | 96 | 252 | 243 |  |  |
| Age (X±sd) | |  |  |  |  |  |  | 0.0178 |  |
|  | 45.47±11.32 | 51.24±13.69 | 45.37±13.57 | 52.94±12.97 | 49.97±13.65 | 47.20±12.44 | 49.85±12.32 |  |  |
| Sex (%) |  |  |  |  |  |  |  | 0.0010 |  |
| Male | 88 (56.41) | 19 (55.88) | 17 (56.67) | 22 (68.75) | 58 (60.42) | 146(41.95) | 175 (72.02) |  |  |
| Female | 68 (43.59) | 15 (44.12) | 13 (43.33) | 10 (31.25) | 38 (39.58) | 106(30.46) | 68 (27.98) |  |  |
| Stage (%) |  |  |  |  |  |  |  |  |  |
| I | - | 1 (2.94) | 15 (50.00) | 1 (3.13) | 17 (17.71) | - | 2 (0.82) |  |  |
| II | - | 7 (20.59) | 0 (0.00) | 3 (9.38) | 10 (10.42) | - | 16 (6.58) |  |  |
| III | - | 4 (11.76) | 3 (10.00) | 8 (25.00) | 15 (15.63) | - | 82 (33.74) |  |  |
| IV | - | 12 (35.29) | 1 (3.33) | 4 (12.50) | 17 (17.71) | - | 105 (43.21) |  |  |
| NA^c^ | - | 10 (29.41) | 11 (36.67) | 16 (50.00) | 37 (38.54) | - | 38 (15.64) |  |  |
| ^a^ Statistical test p-value for comparison between groups of NPC and all control samples; ^b^ Head and Neck Squamous Cell Carcinoma. ^c^ Staging information was not available. | | | | | | | | |  |
|  |  |  |  |  |  |  |  |  |  |
|  |  |  |  |  |  |  |  |  |  |

| **Table S9.** Performance evaluation of EBV DNA methylation detection method. | | | |
| --- | --- | --- | --- |
| Marker | Concentration | CT for Methylated | CT for Unmethylated |
|  | (copies/μL） | products | products |
| Wp | 10^8 | 15.76 | 15.5 |
|  | 10^7 | 19.51 | 19.2 |
|  | 10^6 | 23.33 | 22.68 |
|  | 10^5 | 26.97 | 26.27 |
|  | 10^4 | 30.1 | 29.95 |
|  | 10^3 | 33.38 | 33.18 |
| Cp | 10^8 | 16.59 | 16.72 |
|  | 10^7 | 19.9 | 20.35 |
|  | 10^6 | 23.9 | 24.4 |
|  | 10^5 | 26.63 | 27.46 |
|  | 10^4 | 29.93 | 30.54 |
|  | 10^3 | 32.23 | 33.05 |

| **Table S10.** Primers and probes sequence used in this study. | |
| --- | --- |
| Name | Sequence (5' to 3') |
| BamHI-W-F | CCCAACACTCCACCACACC |
| BamHI-W-R | TCTTAGGAGCTGTCCGAGGG |
| BamHI-W-probe | FAM-CACACACTACACACACCCACCCGTCTC-TAMRA |
| Wp-F | GGGTAGAGATAGGTAGGGT |
| Wp-R | CTCTACCTCCCAAACTTACC |
| Wp-M-probe | FAM-TTTCGAGGAGGCGTTCGGAGTG-BHQ1 |
| Wp-U-probe | HEX-TTTTGAGGAGGTGTTTGGAGTG-BHQ1 |
| Cp-F | GAGTGTTATTTTTGGAATAGTAG |
| Cp-R | TTAAACTCTCTTATTAACTATAATC |
| Cp-M-probe | FAM-TGAATTTTGTTGGCGGGAGAAGGA-BHQ1 |
| Cp-U-probe | HEX-TGAATTTTGTTGGTGGGAGAAGGA-BHQ1 |
